# Impact of left ventricular ejection fraction on the results of cardiac rehabilitation

**DOI:** 10.1007/s12471-019-01305-z

**Published:** 2019-07-08

**Authors:** R. J. G. Peters

**Affiliations:** Department of Cardiology, Academic Medical Center Amsterdam, Amsterdam, The Netherlands

In a wide range of cardiovascular conditions, cardiac rehabilitation is recommended by current guidelines [1, 2]. Ideally, the programme should be initiated before hospital discharge, should commonly continue with a 12-week centre-based programme, and should be maintained indefinitely by the patient. Exercise in a cardiac rehabilitation programme is associated with a low risk of complications and there is wide consensus on the benefits of cardiac rehabilitation. These include reduced mortality, lower risk of rehospitalisation, better control of risk factors, adoption of a healthier lifestyle and improved quality of life [1]. Of note, most of the evidence on these benefits is based on observational studies and most of these studies were published before current therapeutic and preventive options became available [3]. A recent analysis of randomised controlled trials into the effects of exercise-based cardiac rehabilitation versus a no-exercise control, whose participants were recruited after the year 2000, found no effect from cardiac rehabilitation on all-cause mortality or cardiovascular mortality [4]. There was a small overall reduction in hospital admissions. These findings have not yet been incorporated in guidelines. Nonetheless, even if mortality is shown not to be reduced by cardiac rehabilitation in the current era, the other benefits would still support current guideline-based recommendations.

Current guidelines recommend cardiac rehabilitation as a therapeutic intervention in patients with coronary artery disease irrespective of systolic left ventricular function. However, patients with reduced left ventricular ejection fraction (LVEF) are underrepresented in the studies on which these recommendations are based. It is unknown if cardiac rehabilitation is as safe as in patients with a normal LVEF and if the effects of cardiac rehabilitation are similar in both groups of patients. Patients with reduced LVEF appear to be less often referred for cardiac rehabilitation, possibly because of this gap in the scientific evidence [5]. In a very recent meta-analysis, cardiac rehabilitation in patients with reduced LVEF was not associated with a lower rate of clinical events [6]. In this context, the study by Vilela et al., published in this issue of the *Netherlands Heart Journal*, provides highly relevant information [7].

In this observational retrospective cohort study, from a single tertiary centre in Portugal, 379 patients were included after hospitalisation for acute myocardial infarction. Patients were included if they had completed an 8‑week exercise-based cardiac rehabilitation programme that used a Bruce protocol with one-hour sessions. LVEF was estimated by echocardiography (biplane Simpson’s method) and patients were dichotomised by LVEF below or at/above 50%. Peak oxygen uptake (pVO_2_) was measured at baseline and at follow-up and exercise duration was recorded. The results showed that both groups of patients had clear increases in pVO_2_ and in exercise duration. In fact, the proportional increase in pVO_2_ was greater in those with LVEF <50%. Clearly, both groups benefitted in terms of functional improvements, and based on the established association between pVO_2_ and prognosis, the authors suggest that in both groups prognosis was improved. However, clinical events during cardiac rehabilitation or at follow-up were not reported, nor other outcomes such as risk factor management, lifestyle improvements or quality of life.

The findings are consistent with earlier reports that were based on similar observational data. The authors conclude that patients with reduced LVEF have even greater benefits compared with those with a normal LVEF, and this could imply, at least, that patients with reduced LVEF should not be denied cardiac rehabilitation.

Strengths of this study include the high quality cardiac rehabilitation in a tertiary centre, the use of pVO_2_ as a functional outcome measure, and the balanced group sizes. Several limitations need to be addressed. First, there is no information on the characteristics of patients who were not referred for cardiac rehabilitation in spite of having an indication. Second, selection of the patients based on completion of the programme, i.e. exclusion of drop-outs, creates a bias that leads to underestimation of adverse effects. Third, only functional outcomes are reported, whereas several other outcomes may be as relevant in the analysis of the impact of LVEF on the effects of cardiac rehabilitation. In particular, effects on rehospitalisation, adopting regular exercise and quality of life may be influenced by LVEF. Fourth, the 50% LVEF threshold selected by the authors is relatively high, since clinical manifestations of heart failure are likely to develop at lower levels of LVEF. Sixth, as with all previous observations on cardiac rehabilitation, women are underrepresented, with only 19% of patients being female.

In conclusion, the study by Vilela et al. confirms earlier reports that suggest that patients with reduced LVEF derive a functional improvement from cardiac rehabilitation that is at least as great as in patients with normal LVEF. Based on this functional observation alone, however, no conclusions may be drawn on the impact of LVEF on the reduction in clinical events that is one of the major goals of cardiac rehabilitation.
